# The Stemness Gene Mex3A Is a Key Regulator of Neuroblast Proliferation During Neurogenesis

**DOI:** 10.3389/fcell.2020.549533

**Published:** 2020-09-22

**Authors:** Valentina Naef, Miriam De Sarlo, Giovanna Testa, Debora Corsinovi, Roberta Azzarelli, Ugo Borello, Michela Ori

**Affiliations:** ^1^Unità di Biologia Cellulare e dello Sviluppo, Dipartimento di Biologia, Università di Pisa, Pisa, Italy; ^2^Molecular Medicine, IRCCS Fondazione Stella Maris, Pisa, Italy; ^3^Scuola Normale Superiore di Pisa, Pisa, Italy

**Keywords:** neurogenesis, neuroblast, RNA binding protein (RBP), E3 ubiquitin ligase, SOX2, Msi1, Musashi1, Mex3a

## Abstract

Mex3A is an RNA binding protein that can also act as an E3 ubiquitin ligase to control gene expression at the post-transcriptional level. In intestinal adult stem cells, MEX3A is required for cell self-renewal and when overexpressed, MEX3A can contribute to support the proliferation of different cancer cell types. In a completely different context, we found *mex3A* among the genes expressed in neurogenic niches of the embryonic and adult fish brain and, notably, its expression was downregulated during brain aging. The role of mex3A during embryonic and adult neurogenesis in tetrapods is still unknown. Here, we showed that *mex3A* is expressed in the proliferative region of the developing brain in both *Xenopus* and mouse embryos. Using gain and loss of gene function approaches, we showed that, in *Xenopus* embryos, *mex3A* is required for neuroblast proliferation and its depletion reduced the neuroblast pool, leading to microcephaly. The tissue-specific overexpression of *mex3A* in the developing neural plate enhanced the expression of *sox2* and *msi-1* keeping neuroblasts into a proliferative state. It is now clear that the stemness property of mex3A, already demonstrated in adult intestinal stem cells and cancer cells, is a key feature of mex3a also in developing brain, opening new lines of investigation to better understand its role during brain aging and brain cancer development.

## Introduction

In developmental processes, spatial and temporal control of gene expression occurs at transcriptional, post-transcriptional and post-translational levels. More than 1000 genes in the eukaryotic genome encode multifunctional RNA-binding proteins (RBPs) and 50% of these RBPs are expressed in the brain where they regulate all levels of RNA biogenesis at different levels (Bryant and Yazdani, 2016). The neural specific RBPs play a key role in post-transcriptional control, regulating RNA splicing, transport, surveillance, decay and translation (Glisovic et al., 2008).

By RNA-seq analysis we identified a set of evolutionarily conserved, age-regulated genes, expressed in adult neural stem cell niches (aNSCs), in the short-lived fish *Nothobranchius furzeri*, a well-established animal model for aging studies (Baumgart et al., 2014). Among them, the RNA-binding protein mex3A emerged as a putative new neurogenic regulator, down-regulated with age and expressed in neurogenic regions of the zebrafish embryo (Baumgart et al., 2014). This RNA-binding protein belongs to MEX3 family and vertebrates have four distinct mex-3 orthologs (mex-3A–D). All four proteins predominantly accumulate in the cytoplasm, and shuttle between the cytoplasm and the nucleus via CRM1-dependent export pathway (Fornerod et al., 1997). *MEX3* genes encode proteins containing two heterogeneous nuclear ribonucleoprotein K homology (KH) domains and one carboxy-terminal RING finger module with E3 ubiquitin ligase activity (Draper et al., 1996; Buchet-Poyau et al., 2007) sharing the highest identity with *Caenorhabditis elegans* mex-3, a translational repressor involved in the maintenance of germline pluripotency (Ciosk et al., 2006; Hwang and Rose, 2010). The role of *mex3* genes in mammals is poorly understood, though several studies suggest its putative involvement in self-renewal/differentiation decisions with implications for stem cell and cancer biology. In particular, human MEX3A was shown to play a key function in gastrointestinal context by impairing intestinal differentiation and simultaneously promoting an increased expression of intestinal stem cells markers such as *LGR5*, *BML1*, and *MS1* (Pereira et al., 2013, 2020). In mice, *mex3A* is expressed in the crypt base and labels a slowly cycling subpopulation of Lgr5+ intestinal stem cell population (Barriga et al., 2017; Chatterji and Rustgi, 2018). *MEX3A* is overexpressed in pancreatic ductal adenocarcinoma (Wang et al., 2020) and strongly up-regulated in glioblastoma samples (Bufalieri et al., 2020). Despite this evidence, to our knowledge, there are no data available regarding the putative role of *mex3a* during embryonic and adult neurogenesis.

Here we used the clawed frog *Xenopus laevis* embryos to characterize the biological function of *mex3A* in the developing central nervous system (CNS). *Xenopus* embryos gave us the unique opportunity to perform functional experiments in a tissue specific manner without interfering with the normal development of all other tissues (Vitobello et al., 2011; Naef et al., 2018). We showed that *mex3A* is expressed in proliferative regions of *Xenopus* and mouse developing brain including the eye, the brain and neural crest cells. The results from gain and loss of gene function experiments suggested that *mex3A* plays key role in primary mechanisms of proliferation of neural precursors linking cell division and neuronal differentiation during embryonic neurogenesis.

## Materials and Methods

### Molecular Cloning of ***mex3A***

The available Expressed Sequence Tag (EST) clone of *X. laevis mex3A* (ID_6638558, gene bank BC_130195) lacks the coding region at 5′-end. To isolate the 5′-end coding sequence, we used the SMARTTM RACE cDNA Amplification kit (Clontech). The final PCR product was purified and sequenced. We obtained the full-length coding sequence of *X. laevis mex3A* submitted to The National Center for Biotechnology Information (NCBI) (ID_2213511) (Gene bank: MK_800014). A fragment of 975 bp of mouse *mex3a* cDNA (Gene Bank NM_001029890) was amplified and cloned into pGEM-T vector (Promega). The full-length cDNA sequence of zebrafish *mex3a* (Gene Bank XM_009292667) was amplified and cloned into pCS2+ vector.

### Multiple Sequence Alignments of MEX3A Amino Acid Sequences

Multiple sequence alignments of MEX3A amino acid sequences were performed using the NCBI GeneBank for the following organisms: *X. laevis* mex3A (MK_800014); zebrafish mex3a (XM_009292667); *Homo sapiens* MEX3A (NM_001093725.2); *Mus musculus* Mex3A (NM_001029890.2).

### Embryo Collection

Animal handling and care were performed in strict compliance with protocols approved by Italian Ministry of Public Health and of local Ethical Committee of University of Pisa (authorization n. 99/2012-A, 19.04.2012). *X. laevis* embryos were obtained by hormone-induced laying and *in vitro* fertilization then reared in 0.1× Marc’s Modified Ringer’s Solution (MMR 1× : 0.1 M NaCl, 2 mM KCl, 1 mM MgCl2, 5 mM HEPES pH 7.5) until the desired stage according to Nieuwkoop and Faber (Nieuwkoop, 1956).

### Morpholino Oligonucleotides, mRNA *in vitro* Transcription and Microinjections

All morpholinos (MOs) were obtained from Gene Tools, LLC (Philomath, OR, United States). The injections were performed into one side of the embryo in the dorsal blastomere at the 4 cells stage embryo to target neural tissue. The sequences of MOs used were *mex3A MO1* sequence: 5′-CAGCAGG CTCGGCATGGCTAATAAC-3′; *mex3A MO2* sequence: 5′ CATT CCTCTCCATCATCCCTGAGAG-3′; Control Standard Morpholino sequence: 5′-CCTCTTACCTCAGTTACAATTTA TA-3′. Microinjections were performed as described previously (Corsinovi et al., 2019). We injected 12ng per embryos of experimental and control morpholinos. To select properly injected embryos, we co-injected MOs with 250 pg of *gfp* mRNA and we proceeded with the analysis of the embryos that, at neurula stages (stage 15), showed a specific fluorescence in the neural plate of the injected site. The un-injected side represented an internal control in each embryo. We prepared capped *mex3A* and *gfp* mRNAs using the SP6 mMessage Machine *in vitro* transcription kit (Ambion), according to manufacturer’s instructions. For rescue experiments, we co-injected 12ng *mex3A* MO2 and 600ng of full-length *Xenopus* or zebrafish *mex3A* mRNA.

### Whole Mount *in situ* Hybridization

Whole Mount in situ Hybridizations (WISHs) were performed as described (Naef et al., 2018). After color development, embryos were post-fixed and bleached over light to remove the pigment. The following plasmids were used for preparation of antisense RNA probes, enzyme used for linearization and the polymerases used for probe synthesis were and polymerases are indicated; *X. laevis mex3A*-pGEM-T (*Cla*I, Sp6); *pcna-*pBSK, *sox2*-pCS2+, *N-tubulin*-pBKS, *elrC*-pBKS, *huD*-pBSK,; *twist*-pcr2.1 topo (*Hin*dIII,T7); *sox10*-pBKS (*Eco*RI;T3); *slug*-sp72 (EcorV,SP6); *foxd3*-pBSK (*Eco*RI;T7); *msi-1-*pCMV-sport6 (*Eco*RI;T7) (a kind gift from Dr. Romualdo Ciau-Uitz); mouse *mex3A*-pGEM-T (*Not*I, T7).

### *In situ* Hybridization on Frozen Tissue Sections (ISH)

For ISH on cryosections, *Xenopus* embryos were fixed in 4% paraformaldehyde in PBS, cryoprotected with 30% sucrose in PBS and embedded in Tissue-Tek OCT compound (Sakura, 4583). We prepared 12 μm cryosections and ISH was performed according to (Casini et al., 2012). Mouse embryo sections are a kind gift of Prof. Massimo Pasqualetti and were prepared as described in Pelosi et al. (2014). *In situ* Hybridization (ISH) on mouse embryo cryosections at 18 dpc was performed according to (Borello et al., 2014).

### TUNEL and PH3 Staining in *Xenopus* and Statistical Analysis

TdT-mediated DUTP-dig nick end labeling (TUNEL) and PH3 (phospho histone 3) staining were performed according to established protocols (Ori et al., 2006). TUNEL and PH3 positive cells were counted within defined areas in control and injected sides of each manipulated embryo using (Ori et al., 2006) the ImageJ64 software. *P*-values were calculated by paired Student’s *t*-test using GraphPad Prism 6 software (San Diego, CA, United States). Statistical significance was indicated as: ^∗^*p* ≤ 0.05, ^∗∗^*p* ≤ 0.01, ^∗∗∗^*p* ≤ 0.001, ^****^p ≤ 0.0001.

### Measurement of Brain Areas in *Xenopus* and Statistical Analysis

To determine the brain area, embryos at stage 41 (swimming larvae) were anesthetized with buffered tricaine methane sulfonate (MS222) and then fixed in 4% paraformaldehyde in PBS. Brains were isolated using fine forceps and areas of the un-injected and injected sides were calculated using the ImageJ64 software. *P*-values were calculated by paired Student’s *t*-test using GraphPad Prism 6 software (San Diego, CA, United States). Statistical significance was indicated as: ^∗^*p* ≤ 0.05, ^∗∗^*p* ≤ 0.01, ^∗∗∗^*p* ≤ 0.001, ^****^p ≤ 0.0001.

### Quantitative Reverse Transcription Polymerase Chain Reaction and Statistical Analysis

Total RNA was extracted from 30 *Xenopus* morphants at neurula stage (stage 18) using Nucleospin^®^ RNA (Macherey-Nagel) according to manufacturer’s instructions. cDNA was prepared by using iScript^TM^ cDNA Synthesis Kit (Bio-Rad) and quantitative real-time PCR was performed using GoTaq^®^qPCR master mix (Promega) according to the manufacturer’s instruction. Relative expression levels of each gene were calculated using the 2^–ΔΔ*Ct*^ method (Livak and Schmittgen, 2001). The results obtained in three independent experiments were normalized to the expression of housekeeping gene, *gapdh*. The mean of the Control-Morpholino was set at 1. Statistical analysis for qRT-PCR experiments was performed by Student’s *t*-test using GraphPad Prism 6 software (San Diego, CA, United States). Statistical significance was indicated as: ^∗^*p* ≤ 0.05. Following primers were used to perform qRT-PCR: *pcna* (Huyck et al., 2015); *N-tubulin* and *sox2* (De Robertis’s lab, web site: http://www.hhmi.ucla.edu/derobertis/); *elrC* (Seo et al., 2007); Glyceraldehyde 3-phosphate dehydrogenase *(gapdh)* (Naef et al., 2018).

### Statistical Analysis of Embryo Phenotype

Statistical analysis for phenotypes observed after the injection of the Control-Morpholino or the injection of *mex3A*-MO2 was performed by Student’s *t*-test using GraphPad Prism 6 software (San Diego, CA, United States). We compared the percentage of embryos with altered marker genes expression between Control-Morpholino injected embryos and *mex3A*-MO2 injected embryos. Statistical significance was indicated as: ^∗^*p* ≤ 0.5, ^∗∗^*p* ≤ 0.01, ^∗∗∗^*p* ≤ 0.001.

## Results

### Mex3A Is Expressed in the Developing *Xenopus laevis* Brain

**FIGURE 1:**
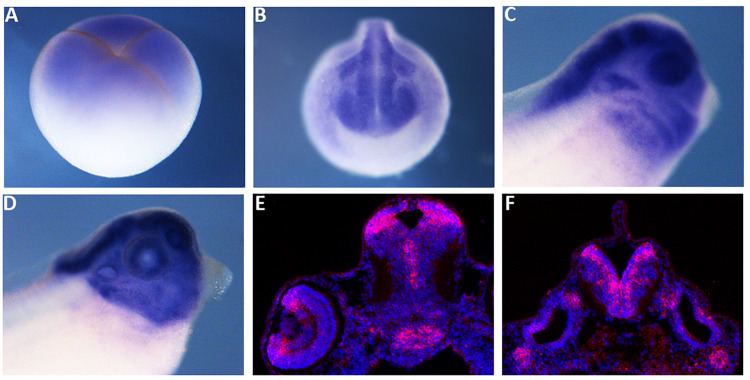
The spatial expression pattern of *Xenopus mex3A*. Whole mount *in situ* hybridization approaches show that *mex3A* is expressed in the central nervous system. **(A)** *Mex3A* expression at blastula stage (stage 3). **(B,C)** At neurula (stage 20) and at tadpole stages (stage 27), *mex3A* is expressed in: neural tube, developing eye, neural crest cells (white arrowhead) and otic vesicle (white arrow). At stage 37, an accumulation of *mex3A* transcript persisted in the most anterior region of the central nervous system **(D)**. **(E,F)** *In situ* hybridization on frozen tissue of transverse sections on WT embryos at stage 41. ISH signal was revealed using fluorescent Fast Red and is visualized in red. Nuclei were revealed in blue with Hoechst. **(E,F)** *Mex3A* is expressed in CMZ of the retina (red arrow), in ventricular zone of the midbrain and in subventricular zone of the hindbrain.

We compared *X. laevis* mex3A predicted protein sequence with the zebrafish, mouse and human homologs revealing a high degree of similarity, especially in RNA binding domains (96%) and C-terminal Ring finger domain with E3 ligase activity (95%) suggesting a conserved function of mex3A in vertebrates (Supplementary Figure 1). As a prerequisite for functional studies, firstly we analyzed the spatial expression pattern of *mex3A* during early embryogenesis. Whole mount *in situ* hybridization (WISH) revealed that *mex3A* is already present in early cleaving stage (four cells stage) before the midblastula transition suggesting that it is maternally supplied (Figure 1A). At mid neurula stage, *mex3A* could be detected in the neural plate, in presumptive eyes territory, in pre-placodal territory and in cranial neural crest cells (NCC) (Figure 1B). At later stages of development, *mex3A* mRNA is present in the eye, in the CNS and in NCC migrated in branchial arches (Figures 1C,D). *In situ* hybridization on cryosections at stage 41 showed the *mex3A* expression in brain areas with high proliferative activity such as the ciliary marginal zone (CMZ) in the retina, the ventricular zone of the midbrain and the subventricular zone of the hindbrain (Figures 1E,F).

### Mex3A Supports Neuroblasts Proliferative State

**FIGURE 2:**
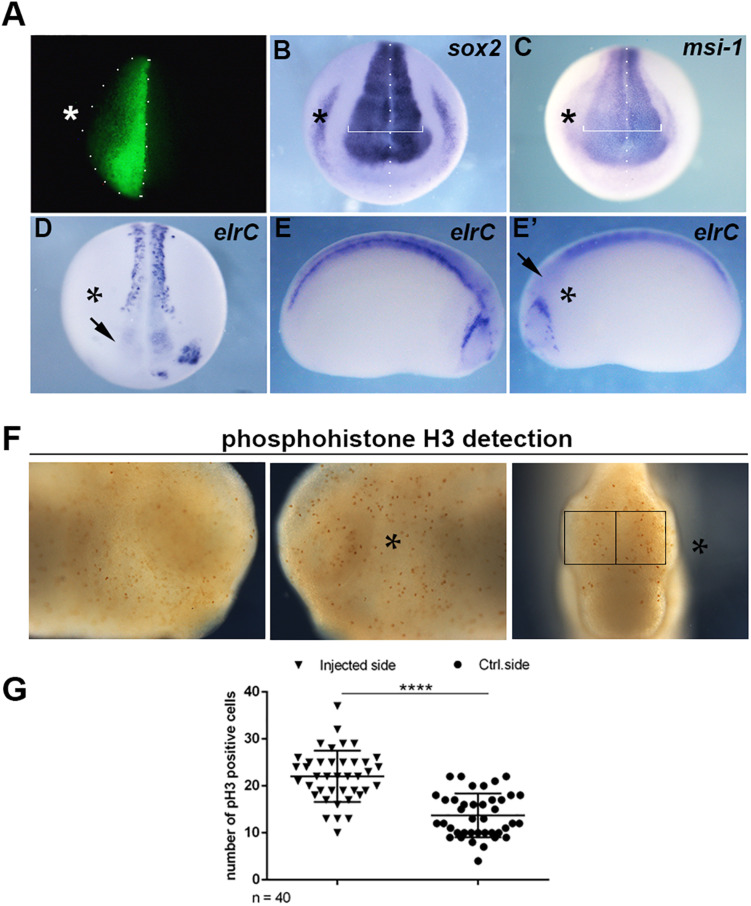
**(A)** Embryos injected with *gfp* (250 pg) and *mex3A* (500 pg) mRNA in one dorsal blastomere at the four-cells stage showing fluorescence only in the neural plate at neurula stage were cultured till different stages of development for WISH analysis. In each panel the asterisk (*) indicates the injected side of the embryo. **(B,C)** mRNA distribution of *sox2* and *msi-1* (*sox2* phenotype 54%, *n* = 116; *msi-1* phenotype 50%, *n* = 80) in *mex3A* overexpressing embryos. The arrow in **(D)** showed the lack of *elrC* expression in the anterior neural plate. **(D–E**′) mRNA distribution of *elrC* at 18 (phenotype 54%, *n* = 114, **D**) and at 23 (phenotype 57%, *n* = 70, **E,E′**) stages in *mex3A* overexpressing embryos. The arrow in **(E′)** shows the lack of neurons in the anterior neural tube. **(F,G)** pH3 positive cells were counted in the areas defined by the black rectangles. Statistical evaluation of the data is shown (*n* = 40). Abbreviations: n total number of processed embryos; error bars indicate standard error of the means (SEM); ***p ≤ 0.001.

Since the expression of *mex3A* suggested a role during primary neurogenesis, we overexpressed *mex3A* in *X. laevis* embryos to evaluate its possible impact on primary neuron formation. For all experiments described below, *mex3A*-mRNA injections were done unilaterally into the animal region of one dorsal blastomere at the four cells stage embryo to target neural tissue. The un-injected side served as internal injection control and the co-injection of *gfp* mRNA was used to select and analyze only embryos in which the transcripts correctly localized in the neural plate (Figure 2A). At neurula stage (stage 18), WISH experiments revealed that the overexpression of *mex3A* altered expression domains of *sox2* and *musashi-1 (msi-1)*. The expression domains of *sox2*, a neuroblast marker (Mizuseki et al., 1998), and *msi-l*, commonly considered a specific marker for stem/progenitor cells (Okano et al., 2005), were markedly expanded in the injected side of the embryo as compared to the un-injected side (Figures 2B,C). Furthermore, we examined the expression of *elrC*, a marker of cells undergoing a transition from proliferation to differentiation (Carruthers et al., 2003), at neurula and tailbud stages. The expression domain of *elrC* appeared dramatically down-regulated in injected side of the embryos compared to un-injected side (Figures 2D–E′). Given these preliminary results, well correlated with the function of human MEX3A as positive regulator of cell cycle progression of intestinal precursors (Pereira et al., 2013; Barriga et al., 2017), we hypothesized that mex3A might be involved in cell proliferation also in the neural context. To elucidate this possibility, we analyzed the number of mitotically active cells in *mex3A* overexpressing embryos by immunostaining for mitotic Ser-10-phosphorylated Histone 3 (pH3). We observed a significant increase in mitotic cell number in the injected side of the embryo compared to the control side (Figures 2F,G). These data suggested that mex3A could maintain the proliferative state of neuroblasts delaying or preventing the neuronal differentiation during embryonic neurogenesis.

### Mex3A Depletion Impairs Primary Neurogenesis

**FIGURE 3:**
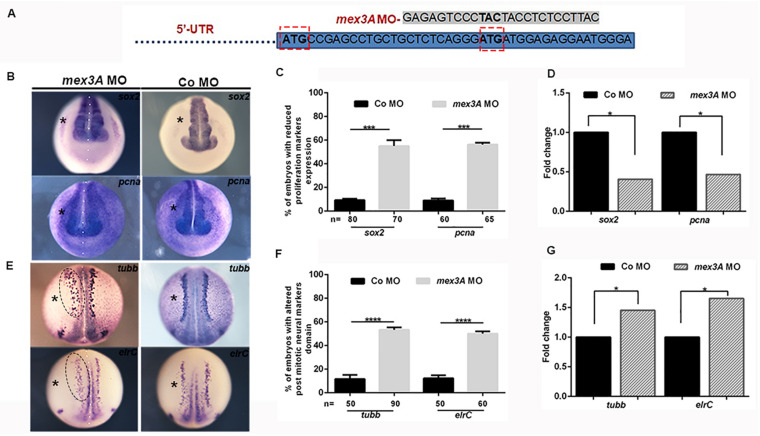
Loss *of mex3A* alters neuronal specification and differentiation **(A)** Structure of *mex3A*-morpholino oligonucleotide. The MO targets the second translation start site. **(B)** mRNA distribution of *sox2* and *pcna* in *mex3A* morphants and controls. **(C)** Quantification of the data in B. **(D)** qRT-PCR analysis. Relative expression levels of each gene are normalized to *gapdh* expression. **(E)** mRNA distribution of *N-tubulin and elrC* in *mex3A* morphants and controls. **(F)** Quantification of the data in **E**. **(G)** qRT PCR analysis. (Abbreviations: *n* number of evaluated embryos in total; error bars indicate standard error of the means (SEM); *p ≤ 0,05, ***p ≤ 0.001, ****p ≤ 0.0001.

To study the role of mex3A in primary neurogenesis context, we also performed experiments of gene loss of function by using a specific morpholino oligo designed to block mRNA translation. However, by analyzing the sequence of the unique *mex3A* exon, we found that there are two possible translation start codons in frame (Supplementary Figure 1). Because both codons can be used as translation initiation sites, if we block the first translation start site using a specific morpholino oligo there is the possibility that the second start site could be used to translate a protein identical to the native one except for the first eight amino acids. The presence of a second ATG in frame and in the same position is conserved in vertebrate orthologs of *mex3A* (Supplementary Figure 1). We designed two specific morpholinos to inject them individually or in combination in the same embryo: morpholino 1 (MO1) designed to block the first ATG and morpholino 2 (MO2) designed to block the second ATG of the *Xenopus mex3A* mRNA. Since the injection of the MO1 did not generate any type of phenotype and the combination of the MO1 and MO2 increased the mortality rate without any synergic or additive effect, we used MO2 alone for subsequent analyses (Figure 3A). A standard control morpholino (CoMO) was used to evaluate non-specific embryo responses. By WISH experiments we showed that the expression domain of *sox2* was reduced in *mex3A*-MO injected side of the embryo whereas both un-injected and CoMO injected sides were unaffected (Figures 3B,C). These data were confirmed by qRT-PCR analysis that showed a significant down-regulation of *sox2* mRNA in *mex3A* morphants (Figure 3D). To further verify whether the loss of *mex3A* function could alter the regulation of neuroblast proliferation, we also examined the mRNA expression of *pcna* (proliferating cell nuclear antigen) (Strzalka and Ziemienowicz, 2011). *Mex3A* morphants showed a reduced *pcna* expression as detected by WISH (Figures 3B,C) and qRT-PCR experiments (Figure 3D). As a consequence of the impairment in the maintenance of neuronal progenitors pool, we observed that the lateral stripe of *N-tubulin* and *elrC* expression domains, the future sensory neurons, appear expanded on the injected side of the embryos compared to control side and CoMO injected embryos (Figures 3E,F). This phenotype might be due to an altered density and/or number of primary neurons. Hence, we performed qRT-PCR analysis that revealed a significant raise of *N-tubulin* and *elrC* mRNA level in *mex3A* morphants (Figure 3G). In order to verify the specificity of the *mex3A-*MO, we designed functional rescue experiments by co-injecting *mex3A*-MO together with the full-length *mex3A* mRNA. As the *mex3A-*MO could target not only the endogenous *mex3A* but also the *in vitro* transcribed *Xenopus mex3A* mRNA, for rescue experiments we cloned the zebrafish *mex3A* mRNA that is not recognized by *mex3A-*MO (Supplementary Figure 3). We already showed that the zebrafish *mex3A* is localized in proliferating region of the developing brain (Baumgart et al., 2014). We further showed that the overexpression of zebrafish *mex3A*, in *Xenopus* embryos, reproduced the same phenotype obtained by the *Xenopus mex3A* mRNA injection, thus confirming its functional conservation (Supplementary Figure 3). We then analyzed 123 co-injected embryos (*mex3A-*MO plus zebrafish mex3A mRNA) and we observed a restoration of the phenotype at neurula stage (stage 18) (Supplementary Figure 3).

### Mex3A Is Required for Anterior Neuronal Development in *Xenopus laevis*

**FIGURE 4:**
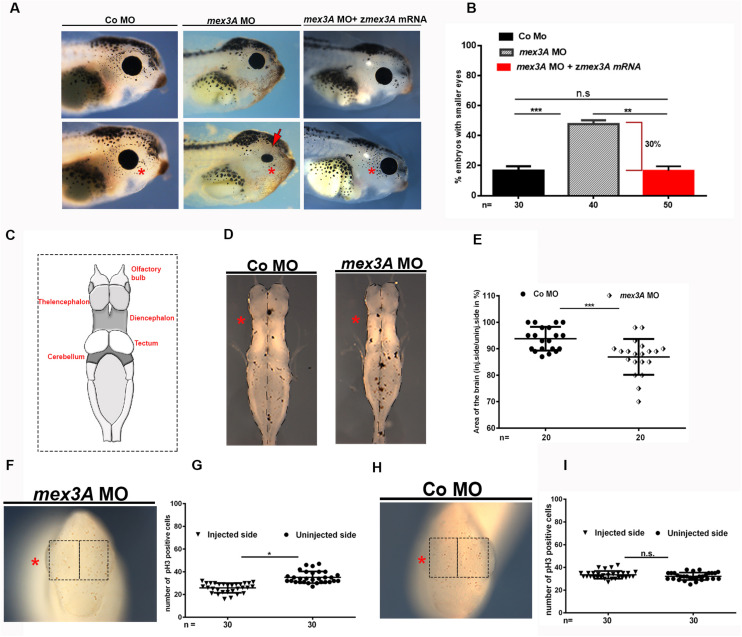
The inhibition of *mex3A* function causes defects in anterior neuronal development. In each panel, the asterisk indicates the injected side of the embryo **(A)** Representative images and **(B)** quantification of the effect of injection of mex3A-MO and co-injection of mex3A-MO with zebrafish *mex3A* mRNA on the eye phenotype. **(C)** Image showing the anatomy of *Xenopus* brain. Diagrams showing a dorsal view of isolated brains. **(D)** Bright field images of *Xenopus* brains at stage 41, anterior to the top after unilateral injection of *mex3A-*MO or CoMO. **(E)** Statistical evaluation of the brain size in injected embryos. **(F–I)** pH3 staining in *mex3A*-deficient embryos at stage 24. *Mex3A* depletion leads to a significant reduction of proliferating cells compared to the un-injected side, whereas the CoMO injection does not influence on proliferation. pH3 positive cells were counted in the areas defined by the black rectangles. Statistical evaluations of the data are shown. Statistical quantifications of the data are given. Abbreviations: *n*, total number of evaluated embryos in total; SEM, error bars indicate standard error of the means; ns, not significant. *p ≤ 0.05; **p ≤ 0.01; ***p ≤ 0.001.

The analysis of gene expression profile of *mex3A* showed a specific *mex3A* expression in the anterior neural tissue in *Xenopus* larvae including eye and brain (Figure 1). Therefore, to investigate in more details the putative biological function of *mex3A* during anterior neural development, we analyzed embryos at later stages of development. We observed in *mex3A* morphants, at larval stage 41, smaller and deformed eye with variable penetrance (Figures 4A,B). In contrast, in control side, as well as in CoMO injected embryos, the eye was always normal (Figure 4A). To test the specificity of *mex3A*-MO to induce eye phenotype, we performed rescue experiments co-injecting the *mex3A*-MO with the zebrafish *mex3A* mRNA, observing a restoration of the eye phenotype (Figures 4A,B). To better show possible alteration in larval brain development, we dissected morphants and control brains from larvae at stage 41 and we measured the areas of both brain hemispheres of injected versus un-injected side. We calculated brain area as described in Kiem et al., 2017. In comparison to the CoMO hemisphere (Figures 4C,D), the *mex3A*-depleted hemisphere exhibited a significant size reduction (Figures 4C,D). This phenotype could be due to a decrease in the cell proliferation rate. To examine this hypothesis, we performed pH3 immunohistochemistry (to visualize mitotic cells) experiments using *mex3A*-depleted embryos at tailbud stage (stage 24). pH3 staining showed a significant reduction in cell proliferation in *mex3A* morphants compared to un-injected control side and to CoMO injection (Figures 4F–I). These results suggested a requirement for mex3A in the control of cell proliferation at both neurula and tailbud stages.

### *Mex3A* Is Expressed in Developing Mouse Brain

The hypothesis that the intestinal stemness-related gene *mex3A* could be considered as a regulator of neuroblast proliferation in the CNS is intriguing but no data are available for the expression of *mex3a* in mammalian CNS. For this reason, we performed a preliminary analysis of mouse *mex3A* mRNA distribution in the developing mouse brain. We revealed that at 18 dpc *mex3A* mRNA is present in proliferating regions of the mouse embryonic CNS such as telencephalic ventricular and sub-ventricular zone, developing hippocampus, olfactory bulbs and olfactory epithelium (Supplementary Figure 2) strongly suggesting a conserved role of *mex3A* in tetrapods CNS.

## Discussion

Mex-3 family members are mediators of post-transcriptional regulation in different organisms (Pereira et al., 2013). Several studies highlighted their involvement in different physiological processes, including the maintenance of the balance between stem cell self-renewal and differentiation. In particular, human MEX3A is necessary to post-transcriptionally regulate the levels of *CDX2*, mRNA coding for an intestinal transcription factor required in gastrointestinal homeostasis (Pereira et al., 2013). Mex3A appears crucial for the maintenance of the slowly cycling subpopulation of lgr5+ gut stem cells (Chatterji and Rustgi, 2018), and lgr5 absence in *Mex3A*^–/–^ mice leads to growth retardation, postnatal mortality, and severe impairment of intestinal crypt development (Pereira et al., 2020).

Recent data showed that MEX3A is up-regulated in glioblastoma specimens (Bufalieri et al., 2020). In glioblastoma cells, MEX3A interacts with the tumor suppressor RIG-I inducing its ubiquitinylation and the proteasome-dependent degradation, supporting tumor growth (Bufalieri et al., 2020). Although MEX3A has a key role in gastrointestinal homeostasis and tumor progression, its putative role in neural context is not yet defined.

Previously, we showed *mex3A* expression in aNSCs niches in *N. furzeri* and in proliferating areas of the developing brain in zebrafish embryos (Baumgart et al., 2014). In the last years, the single cell technologies allowed us to query publicly available datasets and to obtain precious clues on gene expression and possible gene function in different animal models. Transcriptomic analysis of the ventricular-subventricular zone (V-SVZ) of lateral ventricles of male mice at 2, 6, 18, and 22 months revealed *mex3A* among the genes that significantly change their expression, being down regulated, during aging (Apostolopoulou et al., 2017). Benayoun and collaborators included *Mex3A* among the top genes down regulated in olfactory bulbs, another neurogenic niche in the adult brain, during mouse aging (Benayoun et al., 2019). These data nicely correlated with our previous observation of an age-related decline of *mex3a* expression in aNSC niches during *N. furzeri* brain aging (Baumgart et al., 2014) strongly suggesting a functional conservation of the role of mex3a in brain aging among vertebrates. Despite these suggestive clues, nothing is known about mex3A function in the vertebrate nervous system. Here we revealed, for the first time, the expression and function of *mex3A* during early neural development using *X. laevis* as model system. We showed that, besides its widely described role in gastrointestinal context, mex3A is additionally involved in CNS development of tetrapods. *Mex3A* is expressed in the neural tissue of the early *X. laevis* embryo including the eye field and neural crest cells. *Mex3A* mRNA is localized in areas with high proliferative activity such as the ciliary marginal zone (CMZ) of the retina, the ventricular zone of the midbrain and the subventricular zone of the hindbrain strengthening the hypothesis that *mex3A* could promote proliferation of progenitor cells also in neural context. In order to verify possible evolutionary conservation of mex3A role in the developing CNS, we visualized mouse *Mex3A* expression in 18 dpc embryos. We confirmed that *Mex3A* is expressed in proliferative areas of the developing mouse brain, such as in the ventricular-subventricular zone of the lateral ventricles and in the olfactory bulbs. These data suggested a mex3A involvement in the context of primary neurogenesis conserved among vertebrates.

Gene gain and loss of function approaches in *Xenopus* revealed that this gene was able to keep the undifferentiated and proliferative state of neuroblasts increasing the expression of proliferation markers and decreasing the expression of marker such as *elrC (huC)* and *elrD (huD)* during neurogenesis. This evidence suggests that *mex3A* could function as a potential regulator of proliferation rate of neural progenitor cells and this hypothesis is also supported by the increased expression of *musashi-1* in *mex3A* overexpressing embryos. *Msi-1* was first reported to be required for the proper development of the neural sensory organ in *Drosophila* (Nakamura et al., 1994), whereas it is commonly considered a specific marker for stem/progenitor cells in mammals (Kaneko et al., 2000). *Msi-1* maintains stem cell proliferation state by acting as a translational repressor (Ratti et al., 2006). Interestingly, *Msi-1* is regulated by *Mex3A* in mammalian gut cell (Pereira et al., 2013). In *Xenopus* another member of Mex gene family, *mex3b*, is expressed during early development and neurogenesis (Takada et al., 2009). Even if the expression pattern of the *mex3A* and *mex3B* are not overlapping, they seem to be both expressed in the neural plate and then in the neural tube during neurulation. Comparing our data with that obtained by Takada and collaborators, *mex3A* and *mex3B* seem to act not redundantly. The overexpression of *mex3B* in the neuroectoderm did not affect the expression profile of *sox2* (Takada et al., 2009) and the gain or loss of *mex3B* function suggested an involvement of the gene in antero-posterior patterning of the neural tube (Takada et al., 2009). Our results showed that the overexpression, or the knockdown of *mex3A*, did not affect the antero-posterior axis formation or the regionalization of the neural tube supporting the idea that the two genes could act independently and in different time windows during CNS development.

Several neural-specific RNA-binding proteins are key inducers of neuronal proliferation and/or differentiation through the stabilization and/or translational enhancement of target transcripts. Additionally, Mex3A seems to have an important role as post-translational regulators also acting as E3 ubiquitin ligase in glioblastoma cells (Bufalieri et al., 2020).

In conclusion, we showed a key role of *mex3A* as a new post-transcriptional regulator able to influence neuroblast proliferation during neurogenesis. *Mex3A* gene function is necessary and sufficient to support the expression of *sox2* and *msi1*, required for neuroblast self-renewal.

In light of this, in the future, it will be interesting to focus on the possible mex3A targets in neuroblast and adult neural stem cells to better clarify its role in development and aging of the CNS with possible translational implications in brain cancer research.

## Data Availability Statement

The datasets presented in this study can be found in online repositories. The names of the repository/repositories and accession number(s) can be found below: https://www.ncbi.nlm.nih.gov/genbank/, MK800014.1.

## Ethics Statement

The animal study was reviewed and approved by Ministry of Public Health and local Ethical Committee of University of Pisa (authorization n. 99/2012-A, 19.04.2012).

## Author Contributions

VN, MD, and GT performed *Xenopus* experiments. DC and UB cloned mouse mex3a and performed ISH on mouse embryo cryosections. VN and RA contributed in the manuscript discussion and writing. VN performed the data analysis. MO contributed to conceptualization, provided necessary financial resources, experimental supervision, data analysis, discussion, and writing. All authors contributed to the article and approved the submitted version.

## Conflict of Interest

The authors declare that the research was conducted in the absence of any commercial or financial relationships that could be construed as a potential conflict of interest.
