# Supplementary material for: The Stemness Gene Mex3A Is a Key Regulator of Neuroblast Proliferation During Neurogenesis
Source: Front Cell Dev Biol. 2020 Sep 22;8:549533. doi: 10.3389/fcell.2020.549533 (PMC7536324; doi:10.3389/fcell.2020.549533)
Supplement: Supplementary file 1 [file Image_1.PDF]

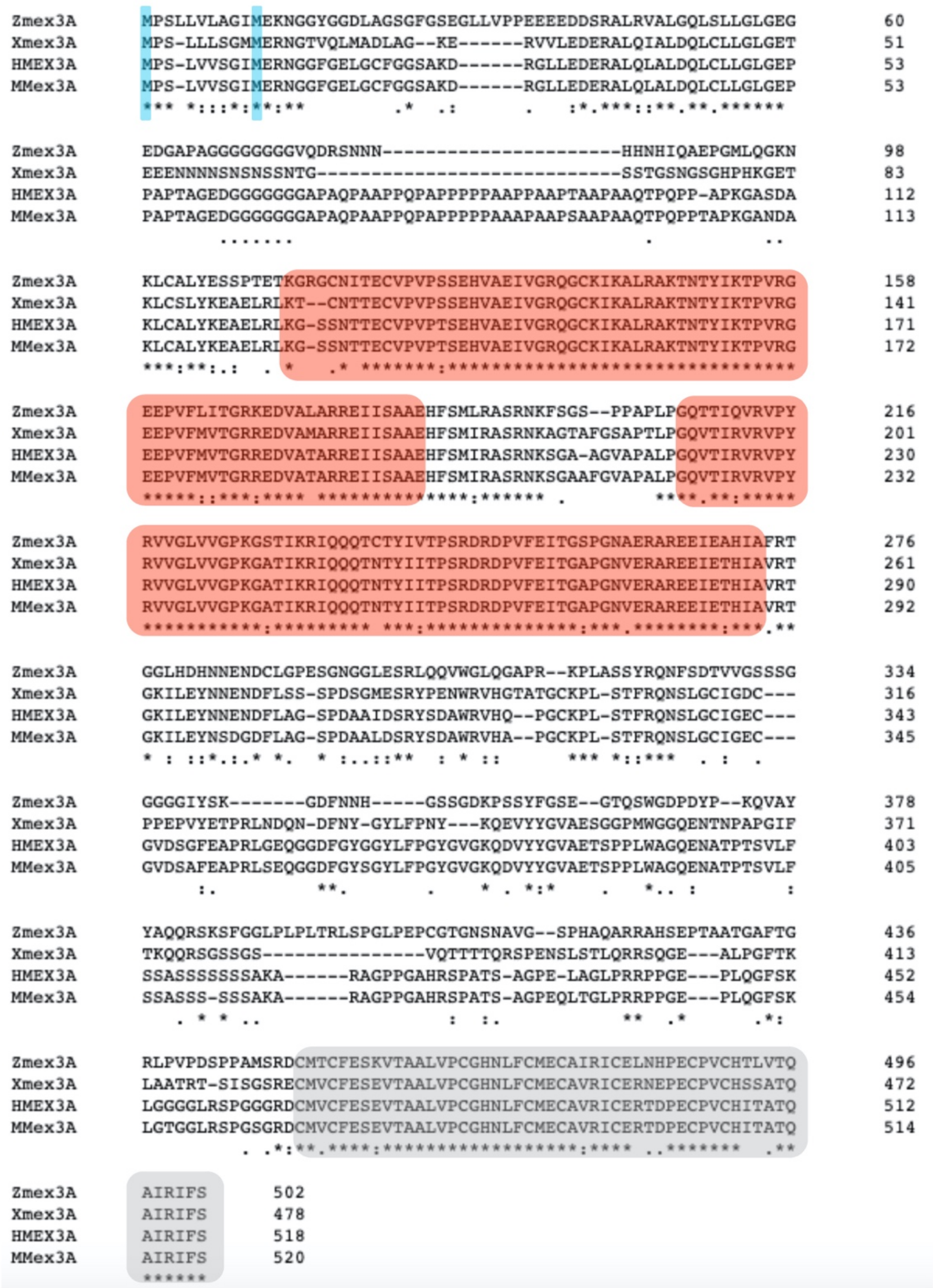

Figure S1. Multiple sequence alignments of Mex3A amino acid sequences.

The blue rectangles highlight two conserved methionine. In red the conserved RNA binding domains (KH) and in grey the C-terminal RING finger domain (RING) with E3 ubiquitin ligase activity of mex3A from Zebrafis (Zmex3A), *X. laevis* (Xmex3A), human MEX3A (HMEX3A) and mouse (MMex3A) obtained using Clustal Omega.
