# Supplementary material for: The Stemness Gene Mex3A Is a Key Regulator of Neuroblast Proliferation During Neurogenesis
Source: Front Cell Dev Biol. 2020 Sep 22;8:549533. doi: 10.3389/fcell.2020.549533 (PMC7536324; doi:10.3389/fcell.2020.549533)
Supplement: Supplementary file 2 [file Image_2.PDF]

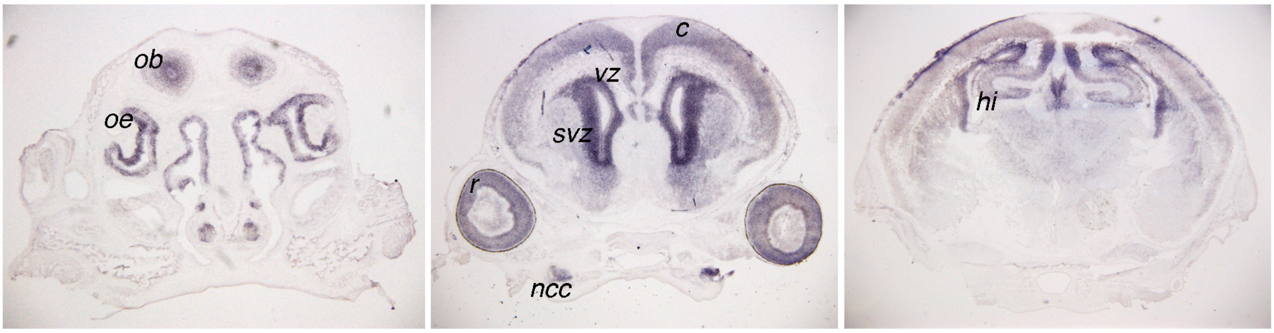

Figure S2. Gene expression of mex3A in E18 mouse embryo.

Coronal cryostat sections of mouse embryo (embryonic day 18) processed by in situ hybridization to visualize mex3a expression pattern. **Ob**, olfactory bulbs; **oe,** olfactory epithelium; **r**, retina; **c** telencephalic cortex; **vz**, ventricular cortical zone; **svz** subventricular zone of basal ganglia; **ncc**, cartilagineous derivatives of neural crest cells; **hi**, developing hippocampal formation.
