# Supplementary material for: The Stemness Gene Mex3A Is a Key Regulator of Neuroblast Proliferation During Neurogenesis
Source: Front Cell Dev Biol. 2020 Sep 22;8:549533. doi: 10.3389/fcell.2020.549533 (PMC7536324; doi:10.3389/fcell.2020.549533)
Supplement: Supplementary file 3 [file Image_3.PDF]

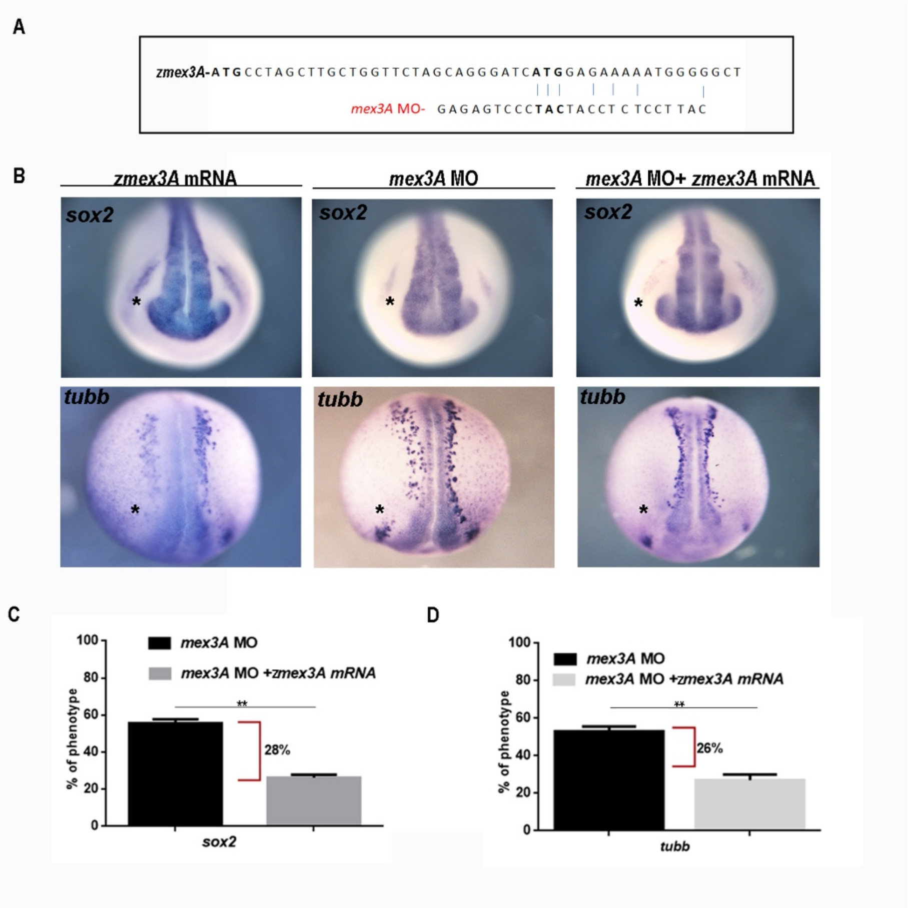

Fig. S3 Functional rescue experiments.

Zebrafish *mex3A* sequence and the mex3A-Mo used in the functional analysis. The morpholino is not able to bind efficiently the zebrafish sequence. The pairing between mex3A-Mo and the zebrafish *mex3A* sequence if affected by 18 mismatches (B) The overexpression of 800pg of zebrafish *mex3A* reproduced the overexpression phenotype observed with the Xenopus *mex3A* mRNA (*sox2* phenotype 52%, n=82; *tubb* phenotype 47%, n=78). In the rescue experiments we injected 12 ng of mex3A-MO and 500 pg of zebrafish *mex3A*. (C, D) The rescue has been calculated on the percentage of embryos in which the correct expression of *Sox2* and *tubb* was restored. (*Sox2* n= 122; *tubb* n=116); error bars indicate standard error of the means (s.e.m); ** p≤0,01.
